# Supplementary material for: CD79A and IL7R mRNA Levels in the Cerebrospinal Fluid of Adults with Acute B-Cell Lymphoblastic Leukemia: A Pilot Study
Source: Diseases. 2025 Jul 1;13(7):206. doi: 10.3390/diseases13070206 (PMC12293166; doi:10.3390/diseases13070206)
Supplement: Supplementary file 1 [file diseases-13-00206-s001.zip › diseases-3602811-supplementary.pdf]

Table S1. Clinical, Molecular Features and Survival Outcome in B-ALL Patients

Code	BM FCM:CD79A	B-ALL subtype	Kariotypes	qPCR LFG	CSF Cytology	CNS3	D/A
1148684	Positive	pre-B	Hyperdiploidy, t(2;5)(q23;p13)[15]/46,XX[10]	Negative	Negative	Negative	D
1146429	Positive	pre-B	Ungrown	Negative	Negative	Negative	A
1170985	Positive	pre-B	Normal	Negative	Negative	Negative	A
1172165	Positive	pre-B	48,XY,−9,add(16)(q24),+3~4mar[4]/47,XY,+11[1]/46,XY[30]	Negative	≤ 5 b/mm³	Positive	A
1182265	Positive	pre-B	Normal	Negative	Negative	Negative	D
1182029	No signal	B common	Normal	Negative	Negative	Negative	A
1182016	Positive	pre-B	46,XX,+5,add(7)(q10),der(14)add(14)(q32),−16,−19,+22[1]/46,XX[29]	Negative	≥ 5 b/mm³	Positive	A
1180010	Positive	pre-B	Norrmal	Negative	Negative	Negative	A
1170637	Positive	pre-B	Ungrown	Negative	Negative	Positive	NT
1163868	Positive	pre-B	Normal	Negative	Negative	Negative	D
1150680	No signal	pre-B	Normal	Negative	Negative	Negative	D
1161154	Positive	pre-B	Ungrown	Negative	Negative	Negative	NT
1162218	Positive	pre-B	Normal	Negative	Negative	Negative	D
1162264	Positive	pre-B	Normal	Negative	≥ 5 b/mm³.	Positive	D
1158854	Positive	pre-B	Normal	*BCR::ABL1*	Negative	Negative	D
1167915	No signal	pre-B	Ungrown	Negative	Negative	Negative	D
1156718	Positive	pro-B	Normal	Negative	Negative	Negative	A
1167121	Positive	pre-B	Normal	Negative	Negative	Negative	D
1163863	positive	pre-B	45,XX,dic,(9;22)(p11~13;q11)[16]/46,XX[4]	Negative	Negative	Negative	D

BM FCM: CD79A, Bone Marrow Flow Cytometry for CD79A; qPCR LFG, qPCR Leukemia Fusion Genes; *BCR::ABL1*, fusion of the BCR and ABL1 genes; D/A, Death or Alive, two-year survival outcome; NT, No tracking; B-subtype classified using French American British (FAB) system and EuroFlow panel; b, blast.

Table S2. RNA Quality and Gene Expression in B-ALL

Code	RNA ng/µl	260/280	Ct CD79A mRNA	Ct IL7R mRNA	Ct GAPDH mRNA	$2^{-\Delta Ct}$ CD79A	$2^{-\Delta Ct}$ IL7R	CNS3
1148684	12.5	1.42	35.5		32	0.088		Negative
1146429	12.3	1.86		29.4	28.4		0.5	Negative
1170985	5.3	1.8			32.7			Negative
1172165	7.1	1.48	32.2	32.9	33.4	2.297	1.414	Positive
1182265	3.5	1.8	29.8		28.4	0.378		Negative
1182029	3.3	1.58			33.7			Negative
1182016	11.8	1.39	31.3	32.4	30.7	0.659	1.32	Positive
1180010	3.8	1.4		30.3	28.4		0.268	Negative
1170637	11.9	2.26	33.4	31	32.6	0.574	3.03	Positive
1163868	3.2	1.29	36		33.5	0.176		Negative
1150680	4.7	1.51	34.8	32	32.5	0.203		Negative
1161154	5.9	1.61	34.5		32.5	0.25		Negative
1162218	3.8	1.79			33.5			Negative
1162264	5.5	1.48	34.9	32.8	32.1	0.143	0.615	Negative
1158854	4.6	1.61	35.1	32	33.6	0.354	2.83	Positive
1167915	7.1	1.52			32.05			Negative
1156718	5.4	1.5			36.9			Negative
1167121	2.9	1.6		35.7	30.5		0.027	Negative
1163863	4.3	1.48			32.8			Negative
